# The Effects of Personality, Anxiety and Depression on the Attentional Processing of Emotional Faces

**DOI:** 10.5334/joc.519

**Published:** 2026-09-10

**Authors:** Carolien Konijnenberg, Svein Åge Kjøs Johnsen

**Affiliations:** 1 Department of Psychology, University of Inland Norway, Norway

**Keywords:** attentional bias, eye tracking, personality traits, anxiety, depression, emotional faces

## Abstract

Individual differences in emotional information processing may be explained by attentional biases related to personality traits and affective symptoms. The present study examined how personality, anxiety, and depression symptoms relate to visual attention toward emotional facial expressions. Participants (N = 103) from a non-clinical student sample completed the Big Five Personality Inventory (BFI) and Hopkins Symptom Checklist (HSCL-25), followed by an eye-tracking task assessing attention to paired neutral and emotional faces. Attentional processing was assessed using initial orienting (time to first fixation) and sustained attention (fixation duration).

Participants showed a general bias toward emotional faces, orienting faster to and fixating longer on emotional compared to neutral stimuli. Higher neuroticism was associated with faster initial orienting to fearful and sad expressions and prolonged fixation on emotional faces overall. Anxiety symptoms were linked to faster initial orienting across emotional expressions, indicating heightened sensitivity to emotional stimuli. Depressive symptoms were associated with longer fixation durations on emotional faces overall, without evidence of mood-congruent bias toward sad expressions. Other personality traits showed limited associations with attentional indices. Gender differences were minimal, although males oriented faster to angry faces.

These findings suggest that neuroticism, anxiety, and depression contribute to distinct components of attentional bias, with neuroticism linked to both early and sustained processing of negative emotional stimuli, anxiety to early vigilance, and depression to prolonged attention to emotional cues. Results support models emphasizing both vigilance and maintenance mechanisms in emotional attention and highlight the role of individual differences in attentional processes.

Individuals differ in how they allocate attention to emotional information, reflecting underlying attentional biases that influence perception and behavior. These biases can be observed as faster detection, prolonged attention, or difficulty disengaging from emotional stimuli (Yiend, 2010). Cognitive factors such as selective attention and working memory contribute to these processes (Wilson & MacLeod, 2003; Xu et al., 2021) as well as personality traits and individual differences in affective style (Ku et al., 2020; Liu et al., 2024).

Personality traits are particularly relevant for understanding variability in attentional bias. Neuroticism has been linked to heightened attention to negative or threatening stimuli, including faster orienting and difficulty disengaging from such cues (Lou et al., 2016; Wang et al., 2022). In contrast, extraversion has been associated with preferential attention to positive stimuli, reflecting sensitivity to rewarding or social cues (Ellingsen et al., 2019). Evidence for other traits, such as agreeableness, conscientiousness, and openness, is less consistent, though they may influence attention to socially relevant information (Pisanu et al., 2024; Wu et al., 2014). In clinical contexts, anxiety is associated with heightened attention to threat-related cues, while depression is linked to a bias toward negative information (Cisler & Koster, 2010; Duque & Vázquez, 2015).

Several theoretical models have been proposed to explain these attentional patterns. The vigilance-avoidance model suggests that anxious individuals rapidly detect and orient attention toward threatening stimuli during early processing stages (Vassilopoulos, 2005). In contrast, the attention maintenance model emphasizes sustained engagement and difficulty disengaging attention from negative information once attention has been allocated (Fox et al., 2001). Finally, the mood-congruent theory proposes that individuals preferentially process stimuli matching their affective state, such as depressed individuals attending to sad cues (Rokke & Lystad, 2015).

Attentional bias has traditionally been studied using reaction-time paradigms such as the dot-probe and emotional Stroop tasks (Lee et al., 2009; Torrence & Troup, 2018). While invaluable for a better understanding of attentional biases, these methods offer only indirect and momentary indices of attention. Eye tracking offers a more direct and continuous assessment, capturing both the allocation and duration of gaze on emotionally salient stimuli (Holmqvist & Andersson, 2017), and is therefore often recommended as a more sensitive and precise measure of visual attention.

Although personality traits and affective symptoms are known to overlap, they are rarely evaluated simultaneously with an eye-tracking paradigm. Examining them together allows for a more comprehensive understanding of whether attentional biases are driven by stable trait disposition (e.g., neuroticism) or affective distress (anxiety/depression). Furthermore, while traditional reaction-time tasks (e.g., dot-probe) capture only momentary attentional snapshots, eye tracking offers a direct and continuous metric of visual gaze, enabling a simultaneous assessment of early orienting and sustained viewing. Combining personality and clinical measures with continuous gaze tracking thus clarifies how trait and state individual differences uniquely shape visual attention.

The present study aims to investigate how personality traits and symptoms of anxiety and depression influence attentional biases toward emotional stimuli using eye tracking. We hypothesized that higher neuroticism and anxiety would be associated with faster orienting and prolonged attention to negatively valenced stimuli (angry, fearful, sad, disgusted), reflecting heightened threat sensitivity and sustained attention to negative cues (Fox et al., 2001; Vassilopoulos, 2005). Depressive symptoms were expected to predict increased attention to sad faces in line with mood-congruent processing (Rokke & Lystad, 2015). In contrast, extraversion was hypothesized to be associated with greater attention to positive stimuli, reflecting an attentional bias toward socially rewarding cues. The study also explores less-studied traits. Agreeableness is expected to be associated with increased attention to happy and sad faces, reflecting sensitivity to positive social cues and empathic concern. Openness and conscientiousness are hypothesized to be associated with more evenly distributed attention across emotional stimuli, consistent with curiosity-driven exploration and systematic goal-directed focus. Gender differences were also examined, with females expected to show stronger biases toward negative stimuli due to higher levels of neuroticism and anxiety (Esplin et al., 2025; Farhane-Medina et al., 2022) and males faster orienting to angry expressions, consistent with findings on gender differences in threat perception (He et al., 2024).

## Methods

### Participants and procedure

A total of 103 students (56 female, 47 male) from a midsize Norwegian university participated. Ages ranged from 18 to 36 years (M = 22.98, SD = 3.51). Students were recruited via convenience sampling during lectures and on campus. Only participants with normal or corrected-to-normal vision (with glasses or contact lenses) could participate to ensure participants could clearly read the self-report measures and perceive the visual face stimuli. A power analysis justifying the sample size is provided in the Supplemental Materials. All participants received written and verbal information about the study and gave written informed consent prior to participation. Testing occurred individually in a quiet room, with participants seated approximately 60 cm from the eye tracker. A five-point calibration preceded the eye-tracking task, followed by two paper-and-pencil questionnaires. Total session time was approximately 20 minutes. Participants were debriefed afterward. No compensation was given. The study was approved by the local research ethics committee (21/01894) and was conducted in accordance with the Declaration of Helsinki.

### Apparatus

Eye movements were recorded using a Tobii Pro Spectrum eye tracker (Tobii Inc., Stockholm, Sweden), at 300 Hz using infrared light. Stimuli were presented on a 23.8-inch integrated monitor. Tobii Pro Lab software was used to present stimuli and process eye-tracking data. Tobii Pro Lab software (version 1.194) was used for presentation.

### Measures

***The Big Five Personality Inventory (BFI)***. The BFI is a survey composed of 44 questions measuring extraversion, agreeableness, conscientiousness, neuroticism, and openness (John & Srivastava, 1999). Respondents indicate the extent to which they agreed with brief statements such as “I see myself as someone who is talkative” on a 7-point Likert scale from 1 (Does not describe me at all) to 7 (Describes me very well). Negative items were recoded and subscale scores were computed by taking the sum of items for each subscale, with higher scores indicating higher levels of the respective trait. The BFI has previously been found both valid and reliable in determining the five key personality traits (John & Srivastava, 1999; McCrae & Costa, 1987). In the present sample BFI subscales demonstrated acceptable to good internal consistency, ranging from α = .73 (Agreeableness) to α = .86 (Neuroticism).

***The Hopkins Symptom Checklist (HSCL-25)***. The HSCL-25 is a self-report measure assessing symptoms of anxiety and depression in both clinical and normative samples (Derogatis et al., 1974). It consists of a 10-item subscale for anxiety and a 15-item subscale for depression. Each item was rated on a 4-point Likert scale (1 = not at all to 4 = very often), referring to symptoms experienced in the past week. Anxiety, depression, and a global symptoms index score was computed as the mean of items on the corresponding subscale or the full scale, respectively. The most-widely suggested cut off score for the HSCL-25 is an average item score of ≥1.75, or a total HSCL-25 score of 43, with scores at or above this threshold indicating elevated symptoms of anxiety and depression (Winokur et al., 1984). The HSCL-25 showed acceptable to excellent internal consistency: α = .79 (anxiety subscale), α = .89 (depression subscale), and α = .91 (full scale), consistent with previous findings (Glaesmer et al., 2014).

**Figure 1:**
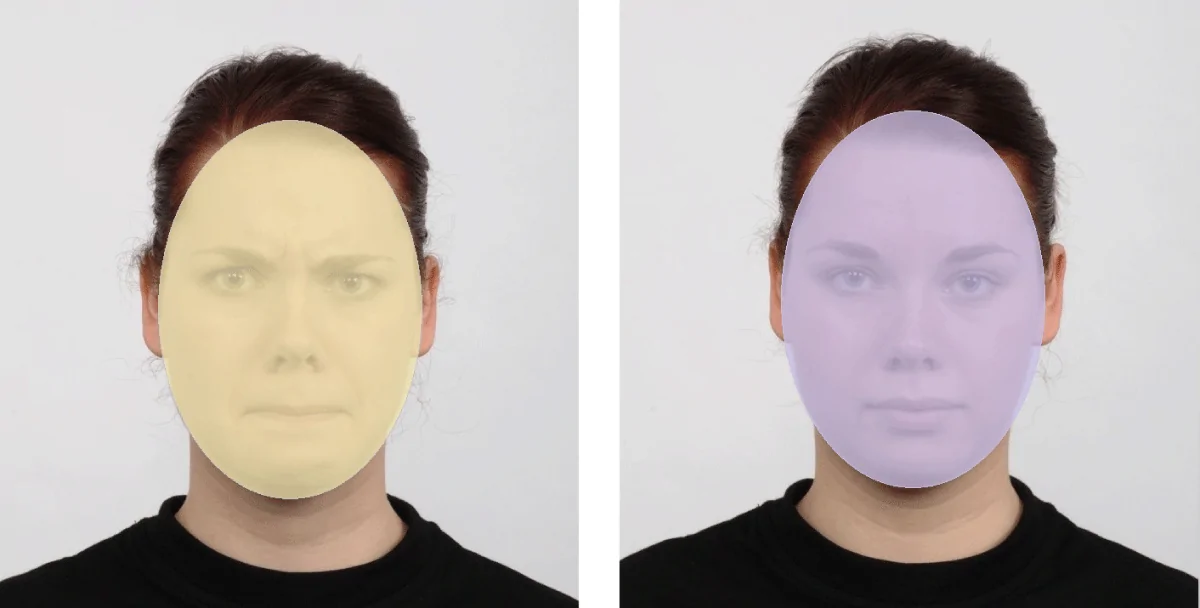
Example trial with a neutral and an emotional expression, presented side by side. Faces were selected from the Radboud Faces Database (RaFD; Langner et al., 2010). Colored outlines indicate face AOIs defined using Tobii Pro Lab. These AOIs were not visible to participants during the experiment but drawn after for analysis.

***Eye tracking task***. Participants viewed 36 trials of paired faces (neutral vs. emotional: angry, disgusted, fearful, happy, sad, surprised) from 6 actors (3 male, 3 female) side by side (see Figure 1 for an example of the trial layout). Neutral face location and trial order were randomized. Each trial began with a small central fixation cross displayed for 500 ms, followed by the two face stimuli for 8 seconds, and an interstimulus interval of 500–1000 ms. Facial stimuli were randomly selected from the validated Radboud Faces Database (RaFD; Langner et al., 2010). Participants were instructed to freely view the faces.

### Data analysis

Data was analyzed using IBM SPSS Statistics software (version 28.0, SPSS, Inc., Chicago, IL). Eye tracking data was analyzed using Tobii Pro Lab analysis software (version 1.194). For each face stimuli, an area of interest (AOI) was drawn around the face (see Figure 1).

The Tobii I-1VT fixation filter was applied to identify fixations. Eye movement samples exceeding a velocity of 30°/s were classified as saccades, whereas movements below this threshold were defined as fixations (Olsen & Matos, 2012). Two eye-tracking metrics were used for each trial: fixation duration, defined as the total time spent fixating within the AOI; and time to first fixation, defined as the latency from stimulus onset to the first fixation on the AOI. For each metric, a bias score was calculated for each participant and for each emotion to quantify attention to emotional faces relative to neutral faces. Fixation duration bias was calculated as the proportion of total fixation time directed at the emotional face relative to the combined fixation duration on both emotional and neutral faces (Fixation duration bias = fixation duration on emotional face/[fixation duration on emotional face + fixation duration on neutral face]). Values greater than 0.5 indicate a bias toward the emotional face (longer fixations), values equal to 0.5 indicate no bias, and values less than 0.5 indicate a bias toward the neutral face. Time to first fixation bias was computed as the difference in latency to first fixation between the neutral and emotional face (Time to first fixation bias = time to first fixation on neutral face – time to first fixation on emotional face). Positive values indicate faster orienting to emotional faces, zero indicates no difference, and negative values indicate faster orienting to neutral faces.

Descriptive statistics were computed for BFI personality traits, HSCL-25 anxiety and depression scores, and eye-tracking measures (fixation duration and time to first fixation) for each emotional expression. Independent-samples t-tests were conducted to examine gender differences in personality, anxiety and depressive symptoms, and eye-tracking measures. To examine attentional bias toward emotional facial expressions, two repeated-measures general linear models (GLMs) analyses were conducted. The first model examined fixation duration bias, and the second examined time to first fixation bias. In both models, emotion (angry, disgusted, fearful, happy, sad, surprised) was specified as a within-subjects factor. Individual differences in personality traits (extraversion, agreeableness, conscientiousness, neuroticism, and openness) and self-reported symptoms of anxiety and depression (HSCL anxiety and HSCL depression scores) were entered as continuous predictors to examine their main effects on attentional bias and their interactions with emotion. Gender was included as a between-subjects factor to account for gender. Parallel mediation analyses (PROCESS Model 4; Hayes, 2017) was used to evaluate whether gender influenced attentional biases indirectly through neuroticism and anxiety. Indirect effects were tested using 5,000 bootstrap resamples; 95% confidence intervals not spanning zero indicated significance. Assumption testing was conducted prior to analysis. Mauchly’s test indicated that the assumption of sphericity was violated for the within-subjects factor emotion, *p* < .001. Accordingly, Greenhouse–Geisser corrections were applied. Pillai’s Trace was used for multivariate tests since Box’s M test was significant, *p* = .02. To ensure model suitability, multicollinearity among continuous predictors was assessed using Variance Inflation Factors (VIF). All VIF values were <3.0 and Tolerance values >0.35, confirming acceptable levels of predictor independence. Effect sizes are reported as partial eta squared (*η*_p_²). The significance level was set at α = .05. These analyses were not formally preregistered. However, the hypotheses, predictors, and analytic approach reported here were determined prior to data analysis and were not modified based on the observed results.

## Results

### Descriptives

Scores on the BFI and HSCL-25, as well as fixation duration and time to first fixation for the different emotional expression in the eye-tracking task are presented in Tables 1 and 2. The mean total score on the HSCL-25 was 46.18 (*SD* = 12.40), with 56 participants (54%) scoring at or above the threshold for elevated symptoms of anxiety and depression. Participants fixated for shorter durations on neutral faces compared to emotional faces and shifted their gaze more quickly to emotional faces than to neutral faces. Mean fixation duration bias values were comparable across emotions, with overall means ranging from .57 to .60, indicating a bias toward emotional faces. Mean time to first fixation bias values ranged from 222 ms to 351 ms across emotions, suggesting faster orientation to emotional faces compared to neutral faces. Intercorrelations among the BFI subscales and HSCL-25 anxiety and depression scores are presented in Table 3.

**Table 1:** Means and standard deviations for Big Five personality traits and HSCL scores.


MEASURE	M (SD)	95% CI

**Big Five Inventory personality trait**		

Extraversion (8–56)	35.67 (8.22)	34.06–37.28

Agreeableness (9–63)	46.53 (7.25)	45.12–47.95

Conscientiousness (9–63)	41.71 (8.73)	40.00–43.41

Neuroticism (8–56)	31.19 (9.33)	29.37–33.02

Openness (10–70)	48.01 (9.40)	46.17–49.85

**HSCL-25**		

Anxiety total (10–40)	18.17 (5.12)	17.17–19.17

Depression total (15–60)	28.01 (8.46)	26.36–29.66

Global Symptom Index (25–100)	46.18 (12.40)	43.76–48.61


*Note*. HSCL-25 = Hopkins Symptom Checklist-25.

**Table 2:** Means and standard deviations for eye-tracking measures by emotion.


MEASURE	M (SD)	95% CI

**Eye-tracking: Fixation duration (ms)**		

Neutral	2324 (556)	2215–2433

Angry	3186 (915)	3007–3365

Disgusted	3163 (849)	2997–3329

Fear	3224 (875)	3053–3396

Happy	3462 (785)	3308–3615

Sad	3297 (832)	3135–3460

Surprised	3288 (856)	3121–3455

**Eye-tracking: Time to first fixation (ms)**		

Neutral	1027 (416)	946–1108

Angry	712 (368)	640–784

Disgusted	676 (342)	609–743

Fear	728 (398)	650–805

Happy	773 (477)	680–866

Sad	804 (537)	699–909

Surprised	685 (328)	621–750


**Table 3:** Intercorrelations for BFI subscales and HSCL-25 scores.


	1	2	3	4	5	6	7

1. Extraversion	—						

2. Agreeableness	.23*	—					

3. Conscientiousness	–.01	.11	—				

4. Neuroticism	–.30**	–.34**	–.43**	—			

5. Openness	.03	.03	–.08	.04	—		

6. HSCL-25 Anxiety	–.05	–.34**	–.27**	.56**	–.06	—	

7. HSCL-25 Depression	–.17	–.25*	–.42**	.63**	.14	.65**	—


*Note*. HSCL-25 = Hopkins Symptom Checklist-25. *p < .05. **p < .01.

On the BFI, females scored higher on neuroticism than males (M = 33.64, SD = 8,96 and M = 28.28, SD = 8.99, respectively), *t*(101) = –3.02, *p* = .002, 95% CI [–8.89, –1.84]. No other personality traits differed between males and females (all *p*s > .05). A gender difference was also observed in anxiety symptoms, with females reporting higher scores than males on the HSCL-25 anxiety scale (M = 19.25, SD = 5.31 and M = 16.89, SD = 4.61, respectively), *t*(101) = –2.38, *p* = .010, 95% CI [–4.32, –0.39], but not in depressive symptoms, *p* > .05. No gender differences were found for fixation duration or time to first fixation, with the exception of time to first fixation on angry faces, where males were faster than females to orient to the angry face (M = 624.39 ms, SD = 308.55, vs. M = 785.17 ms, SD = 398.84, respectively), *t*(101) = –2.25, *p* = .013, 95% CI [–302.27, –19.30].

### Fixation duration bias

Results from the repeated-measures GLM showed no significant main effect of emotion on fixation duration bias, Pillai’s Trace = .02, *F*(5, 90) = 0.29, *p* = .92, *η*_p_² = .02, indicating that the tendency to fixate on emotional faces was comparable across all emotional expressions. None of the interactions between emotion and personality traits or HSCL-25 anxiety and depression scores were significant (all *p*s > .05), suggesting that their effects on fixation duration bias were consistent across emotions. When averaged across emotions, neuroticism significantly predicted fixation duration bias, *F*(1, 94) = 4.31, *p* = .04, *η*_p_² = .04, with higher neuroticism scores associated with longer viewing time on emotional faces relative to neutral faces. Similarly, HSCL-25 depression scores significantly predicted fixation duration bias, *F*(1, 94) = 5.18, *p* = .03, *η*_p_² = .05, with higher depression scores associated with greater attention to emotional faces. Extraversion, agreeableness, conscientiousness, openness, and HSCL-25 anxiety were not significantly associated with fixation duration bias (all *p*s > .05).

Gender was not significantly associated with fixation duration bias, *F*(1, 94) = 0.03, *p* = .86, *η*_p_² < .001. To examine whether gender influenced fixation duration bias indirectly through neuroticism and anxiety a parallel mediation analysis (PROCESS Model 4, 5,000 bootstrap resamples) was conducted. Results indicated no significant total effect (c = 0.007, *p* = .70, 95% CI [–0.030, 0.045]) or direct effect (c’ = 0.001, *p* = .89, 95% CI [–0.036, 0.041]) of gender on fixation duration bias. The indirect effects through neuroticism (*ab* = 0.013, 95% CI [–0.001, 0.034]) and anxiety (*ab* = –0.008, 95% CI [–0.030, 0.006]) were also non-significant, as both 95% intervals included zero.

### Time to first fixation bias

The multivariate test investigating time to first fixation bias showed no significant main effect of emotion on time to first fixation bias, Pillai’s Trace = .08, *F*(5, 90) = 1.66, *p* = .15, *η*_p_² = .08, suggesting that initial orienting bias toward emotional versus neutral faces did not differ across emotional expressions. Within the GLM, tests of between-subjects main effects showed that both neuroticism and HSCL-25 anxiety significantly predicted time to first fixation bias, with neuroticism, *F*(1, 94) = 6.97, *p* = .010, *η*p² = .07, and anxiety, *F*(1, 94) = 4.33, *p* = .040, *η*_p_² = .04, associated with faster overall orienting toward emotional faces. Extraversion, agreeableness, conscientiousness, openness, and HSCL depression were not significant predictors (all *p*s > .05).

There was a significant interaction between emotion and neuroticism, Pillai’s Trace = .18, F(5, 90) = 4.03, p = .002, *η*_p_² = .18 and between emotion and agreeableness, Pillai’s Trace = .14, F(5, 90) = 2.86, *p* = .02, *η*_p_² = .14. No other interactions were significant (all *p*s ≥ .26). To further investigate these two significant interactions and determine which specific emotional expressions participants oriented towards, follow-up univariate tests and an examination of parameter estimates were conducted for each emotion. Follow-up analyses were evaluated against a Bonferroni-corrected threshold of α = .0083 to account for comparisons across the six emotional expressions. Results indicated that neuroticism was significantly associated with faster orienting toward fearful, *B* = 24.11, *SE* = 7.49, *p* = .002, *η*_p_² = .10 and sad faces, *B* = 33.90, *SE* = 9.75, *p* < .001, *η*_p_² = .11. In both cases, higher neuroticism scores were associated with shorter latencies to first fixation on emotional faces relative to neutral faces. Neuroticism was not significantly related to orienting bias for angry, happy, disgusted or surprised faces (all *p*s > .0083). For the interaction involving agreeableness, no single emotional expression reached statistical significance under Bonferroni-corrected thresholds (all *ps* > .0083), despite a trend toward faster orienting to fearful faces, *B* = 15.92, *SE* = 6.95, *t*(94) = 2.29, *p* = .02, *η*_p_² = .05.

Gender was not significantly associated with time to first fixation bias, *F*(1, 94) = 0.25, *p* = .62, *η*_p_² = .003. To evaluate whether neuroticism and anxiety explained the relationship between gender and initial orienting bias, a parallel mediation analysis (PROCESS Model 4, 5,000 bootstrap resamples) was conducted. While the total effect (c = 7.59, *p* = .91, 95% CI [–140.0, 155.1]) and direct effect (c’ = 0.08, p = .99, 95% CI [–147.1, 148.7]) of gender were non-significant, significant opposing indirect effects emerged. Female gender was indirectly associated with faster orienting to emotional faces through higher neuroticism (*ab* = 68.91, 95% CI [6.74, 161.28]) but with slower orienting to emotional faces through higher anxiety (*ab* = –62.12, 95% CI [–165.94, –1.01]).

## Discussion

The present study investigated how personality traits and symptoms of anxiety and depression were associated with attentional bias toward emotional faces. Neuroticism predicted longer viewing time on emotional faces, irrespective of their valence, and faster initial orienting toward negatively valenced emotional stimuli (fearful and sad), but not happy or surprised faces. These findings align with theoretical models emphasizing both early vigilance and sustained processing of threat-related information (Fox et al., 2001; Vassilopoulos, 2005). Results are also consistent with earlier eye-tracking research linking neuroticism to increased attention to fearful expressions (Perlman et al., 2009) and a more recent meta-analysis showing associations between anxiety and fear- related symptoms and both vigilance toward and maintenance of attention on threat cues (Clauss et al., 2022). These findings suggest neuroticism to be associated with heightened sensitivity to aversive and distress-related emotional cues.

Anxiety symptoms were associated with faster initial orienting toward emotional faces but not with prolonged fixation. This pattern suggests that anxiety primarily influences early attentional processes rather than sustained attention, consistent with vigilance-based accounts (Vassilopoulos, 2005). However, this effect was observed across all emotional expressions, not only toward threat cues (angry/fearful faces), indicating a general sensitivity to emotional salience rather than a threat-specific bias in this non-clinical sample.

Depressive symptoms were associated with increased fixation on emotional faces overall, rather than specifically to sad faces. This finding does not support the mood-congruent hypothesis predicting depressive symptoms to be associated with increased attention to sad emotional stimuli (Rokke & Lystad, 2015). Instead, depressive symptoms were associated with a generalized increase in attention to emotional stimuli. This broader pattern may reflect heightened emotional reactivity, whereby emotional stimuli become more salient regardless of content, or reduced attentional selectivity, where depressive symptoms impair the ability prioritize specific emotional categories. This differs from clinical samples, where a valence-specific bias toward dysphoric and sad stimuli, alongside reduced attention to positive stimuli has been found (Suslow et al., 2020). Such valence-specific bias may only emerge at higher symptom severity, not yet detectable in subclinical samples like ours.

Contrary to expectations, extraversion was not associated with increased attention to positive stimuli, contrasting with previous findings (Ellingsen et al., 2019). This discrepancy may be due to methodological differences, as Ellingsen et al. (2019) only compared individuals with the 10 highest and 10 lowest extraversion scores which may have amplified trait-related differences in attentional patterns. Similarly, agreeableness showed limited effects. While agreeableness showed a general relationship with how people looked at the different emotions, this effect did not map onto any single emotional expression under closer examination. Nevertheless, a subtle pattern emerged suggesting a tendency to look more quickly at fearful faces. If this pattern holds true, it may reflect a heightened sensitivity to socially relevant threat cues rather than general empathic or affiliative processing. Fearful expressions signal potential environmental danger and are socially informative, which may engage individuals who are attentive to interpersonal dynamics. No effects were observed for conscientiousness or openness, suggesting that these traits may play a limited role in the allocation of attention to emotional stimuli in passive viewing contexts.

The lack of overall main effects for emotion, as well as the absent associations for traits like extraversion and openness, suggests that attentional biases toward emotional faces in non-clinical samples may be more generalized or subtle than traditional valence-specific models propose. Rather than strong category-specific biases, individual differences in this population appear to operate mainly through general emotional salience and broad threat sensitivity.

Gender differences were minimal. Although females reported higher levels of neuroticism and anxiety, this did not translate into stronger attentional biases. For initial orienting, higher neuroticism pulled attention faster toward emotional faces, but this was counterbalanced by anxiety’s slowing effect, leaving total gender differences near zero. However, males did orient faster to angry faces, consistent with previous findings on threat processing (He et al., 2024).

Several limitations should be noted. The sample consisted of university students, limiting generalizability. Since attentional patterns toward emotional stimuli can vary with age, these findings may be most representative of younger adults. Anxiety and depression were assessed using self-report measures, making them susceptible to recall bias, response styles, differences in interpretation, and situational influences. Although not drawn from a clinical population, participants scored relatively high, above the threshold for elevated anxiety and depression symptoms, indicating a relatively symptomatic, subclinical sample. Findings may therefore not fully generalize to low-symptom community samples, nor to clinical populations where symptom severity is typically greater. It also means the observed associations, including the absence of a valence-specific mood-congruent bias, were detected within an already elevated-symptom sample, so it remains unclear whether this pattern reflects a genuine absence of mood-congruent bias at a subclinical level, or would shift toward a more valence-specific bias at higher symptom severity. Additionally, effect sizes were modest, suggesting that attentional bias is influenced by multiple factors beyond personality traits and anxiety and depression symptoms. Another limitation is that difference-score bias metrics in attentional research often exhibit lower reliability when assessing individual differences, which can attenuate observed correlations. Finally, while total fixation duration provides a robust measure of overall visual engagement and sustained attention, it does not directly isolate disengagement mechanisms, findings should therefore be interpreted as reflecting broad attentional engagement rather than specific disengagement deficits.

The present findings are consistent with the idea that attentional bias plays a role in emotional vulnerability. Given that our data comes from a non-clinical sample and are purely correlational, it would be premature to conclude that attentional bias plays a causal role in the manifestation of anxiety and depressive symptoms. Nonetheless, the observed patterns, prolonged attention toward negative stimuli among individuals high in neuroticism, and faster orienting toward emotional stimuli among those higher in anxiety, align with the mechanisms targeted by attention-bias modification (ABM) tasks such as the dot-probe paradigm (MacLeod et al., 2002). Given the study’s limitations, however, these findings are best as informing future intervention research rather than as direct support for clinical application, particularly as meta-analytic evidence on the effects of ABM remains uncertain (Fodor et al., 2020).

To conclude, the present study demonstrates that neuroticism, anxiety and depression symptoms are associated with distinct aspects of attentional biases toward emotional faces. Neuroticism was linked to both early and sustained attention to negative valenced expressions, anxiety to faster initial orienting to all emotional stimuli, and depressive symptoms to increased overall attention to all emotional expressions. Other personality traits showed limited influence. These findings highlight the importance of individual differences in shaping attentional patterns and inform targeted interventions for regulating emotional reactivity.

## Additional File

The additional file for this article can be found as follows:

10.5334/joc.519.s1 Supplementary Materials. Additional methodological details including post-hoc power analysis and eye-tracking data quality metrics.

## Data Availability

The dataset supporting the findings of this study is openly available in Zenodo at https://doi.org/10.5281/zenodo.22640075.
