# Supplementary material for: The Effects of Personality, Anxiety and Depression on the Attentional Processing of Emotional Faces
Source: J Cogn. 2026 Sep 10;9(1):46. doi: 10.5334/joc.519 (PMC13565027; doi:10.5334/joc.519)
Supplement: Supplementary file 1 [file joc-9-1-519-s1.pdf]

Power analysis

We conducted a post-hoc power analysis using G*Power (v3.1.9.7) for our GLMs to check the adequacy of our sample size (n = 103). For the repeated-measures GLMs with 6 measurements and an alpha level of .05, a statistical power of .9997 for our smallest observed significant effect size ($\eta p^2 = .04$, equaling $f = 0.204$) was found. With a standard medium effect size of f = 0.25, our power is nearly 100% (.9999), demonstrating that our study was robustly powered to detect moderate effects. While a larger sample size (e.g., n > 350) might theoretically push very small effects ($\eta p^2 = .01$) into statistical significance, such an effect would most likely hold limited practical or theoretical relevance. The lack of significance for extraversion, conscientiousness, and openness suggests their true effects in the current study are either non-existent or very small.

Eye tracking data quality:

To ensure high spatial data quality, a 5-point calibration and a 4-point validation procedure were performed in Tobii Pro Lab for each participant. Across the entire sample (n=103) the mean validation accuracy (spatial calibration error) was *M* = 0.58, *SD* = 0.38°, min = 0.18°, max = 3.11°. The total amount of data loss was also investigated, the mean percentage of successfully captured gaze samples was M = 91%, SD = 6.30, corresponding to an average data loss of 9%. At the trial level, an individual trial was excluded if the participant failed to look at either of the two face stimuli (i.e. had no gaze fixations on the face AOI) at any point during the 8 second presentation. At the participant level, we set an inclusion criterion requiring a minimum of 27 valid trials (75% of the 36 trials) to be retained in the final dataset. All 103 participants met this criterion, therefore, no participants were excluded from the final analysis. Split-half reliability of the two attentional bas indices was estimated by dividing the six face identities into two parallel halves and correlating the mean bias scores derived from each half across participants. Fixation duration bias showed excellent split-half reliability (Spearman–Brown corrected $r = .92$, $p < .001$). Time to first fixation bias showed weaker reliability (Spearman–Brown corrected $r = .41$, $p = .007$). Lower reliability for latency-based difference scores relative to proportion-based measures is a common pattern in eye-tracking research, likely reflecting that time to first fixation is determined by a single gaze event per trial rather than being aggregated across the full viewing period, as fixation duration is.
